# High-Temperature Hydrofluoric Acid Etching Increases the Debonding Resistance of Zirconia Copings Cemented to Titanium Bases: An In Vitro Study

**DOI:** 10.3390/ma19061191

**Published:** 2026-03-18

**Authors:** Sara Varas-Orozco, Esteban Pérez-Pevida, Jordi Martínez-López, José Manuel Mendes, Javier Gil-Mur, Aritza Brizuela-Velasco

**Affiliations:** 1 Bioengineering Institute of Technology, Faculty of Medicine and Health Sciences, International University of Catalonia, 08195 Sant Cugat del Valles, Spain; saravarasorozco@gmail.com; 2 DENS-ia Research Group, Faculty of Health Sciences, Miguel de Cervantes European University, 47012 Valladolid, Spain; abrizuela@uemc.es; 3 Department of Surgery, Faculty of Medicine, University of Salamanca, 37008 Salamanca, Spain; 4 Soadco Research and Development Department, AD700 Escaldes, Andorra; j.martinez@soadco.com; 5 UNIPRO—Oral Pathology and Rehabilitation Research Unit, University Institute of Health Sciences, Cooperative of Polytechnic and University Higher Education (CESPU), 4585-116 Gandra, Portugal; jose.mendes@iucs.cespu.pt; 6 Bioinspired Oral Biomaterials and Interfaces, Department of Materials Science and Engineering, Polytechnic University of Catalonia, 08019 Barcelona, Spain; javier.gil.mur@upc.edu

**Keywords:** zirconia, Ti-base, cementation, hydrofluoric acid etching, retention strength, thermocycling

## Abstract

**Highlights:**

- This protocol represents an effective surface-conditioning strategy to improve the stability of zirconia copings cemented to titanium bases.
- Potentially reduce the risk of clinical debonding.

**Abstract:**

This study compared three internal surface treatments of zirconia copings—silane alone (control), airborne-particle abrasion followed by silane, and high-temperature hydrofluoric acid etching followed by silane—regarding initial pull-out retention strength, retention after thermocycling, failure mode assessed by scanning electron microscopy (SEM), and surface wettability. Sixty-three monolithic zirconia copings were allocated to three groups (n = 21) according to surface treatment and cemented to titanium bases with a self-adhesive resin cement. Initial pull-out tests were performed. A subset (n = 10 per group) underwent thermocycling followed by repeat testing. Failure modes were analysed by SEM, and wettability was measured using the sessile drop method. Surface roughness and crystalline phase were additionally characterized by white-light interferometry and X-ray diffraction (XRD), respectively. High-temperature acid etching produced significantly higher initial pull-out forces than airborne-particle abrasion and silane alone, with mean values 125% higher than control and 42.6% higher than airborne-particle abrasion. After thermocycling, acid-etched specimens maintained the highest retention, whereas airborne-particle abrasion showed critical loss. SEM revealed predominantly cement remnants on zirconia in the acid-etched group, indicating a stronger zirconia–cement interface. Acid etching also yielded significantly lower contact angles, reflecting improved wettability. High-temperature hydrofluoric acid etching followed by silanization provided superior and more stable retention, more favourable failure modes, and improved wettability.

## 1. Introduction

All-ceramic dental restorations have become a well-established alternative to conventional metal–ceramic restorations due to their excellent biocompatibility, mechanical properties, and favourable aesthetics [1]. Among these materials, yttria-stabilised tetragonal zirconia polycrystal (Y-TZP), commonly referred to as zirconia (Zr), is notable for its high compressive strength. Nevertheless, zirconia has certain limitations under shear loading, which restrict its direct clinical use as an implant abutment. Several studies have reported that fracture of titanium abutments is clinically infrequent (0.08% at 5 years), whereas fracture rates for zirconia abutments may reach up to 1.8% at the same follow-up interval [2,3,4].

For this reason, the use of specific titanium abutments (Ti-base) has become widespread. These abutments serve as a platform for a customised zirconia restoration, which is cemented onto the Ti-base and subsequently screw-retained to the implant [5]. However, one of the most frequent technical complications associated with this prosthetic solution is the loss of retention of zirconia crowns cemented onto Ti-bases, with reported rates of up to 5% at 5 years. Debonding is not a trivial event: it may require complete recementation or even fabrication of a new restoration, compromise occlusal stability, lead to additional appointments, increase treatment costs, and—when microleakage is present—favour biological complications such as peri-implant inflammation [6,7].

Although debonding of cemented or screw–cement-retained zirconia crowns and bridges is relatively common, the available information regarding cementation protocols and their effect on retention remains limited. Authors differ in their recommendations regarding the type of cement: provisional and permanent cements have been used, including zinc phosphate, glass ionomer, and resin cements, with the latter—particularly self-adhesive resin cements—showing superior retention [8,9].

Another relevant line of investigation concerns the influence of zirconia surface treatment on improving adhesive performance. These treatments may be mechanical, such as airborne-particle abrasion or laser modification, which increase micromechanical retention, or chemical, such as the application of silane agents, glazing, or acid etching. Mechanical treatments can promote adhesion between zirconia and resin cement; for example, airborne-particle abrasion with silica-coated alumina particles enables subsequent silanization [10,11]. However, abrasion may deteriorate the mechanical properties of zirconia by inducing surface defects and facilitating tetragonal-to-monoclinic phase transformation, which can generate microcracks and compromise the long-term stability of the restoration [12,13].

In contrast, chemical treatments such as acid etching increase surface roughness and create micromechanical undercuts without substantially altering the structural properties of zirconia [14,15,16]. Nevertheless, there is controversy surrounding conventional hydrofluoric acid etching at room temperature. Several authors have suggested that increasing the temperature, together with high-concentration solutions and prolonged exposure times, may allow effective etching of zirconia surfaces, although evidence remains limited [17].

Regarding the chemistry of adhesion, silanes are bifunctional molecules capable of chemically coupling inorganic silica-containing substrates with organic resin matrices. Because zirconia contains no silica, conventional silanization is ineffective; therefore, zirconia-specific primers incorporating functional monomers such as 10-MDP (10-methacryloyloxydecyl dihydrogen phosphate) have been developed. These monomers can establish stable bonds with metal oxides on the zirconia surface following appropriate activation or surface modification.

Another aspect that remains insufficiently explored is the relationship between surface treatment, wettability, and the resulting pattern of adhesive failure. Wettability directly influences the ability of resin cement to spread and penetrate into the created surface irregularities, while failure-mode analysis using scanning electron microscopy (SEM) provides additional insight into the integrity of the Zr–cement–Ti interface. Only a limited number of studies have integrated, within the same experimental model, mechanical retention testing, artificial aging, SEM evaluation, and contact-angle measurement.

For these reasons, the aim of this in vitro study was to compare three internal surface treatments of zirconia copings (silane application alone, airborne-particle abrasion followed by silane, and high-temperature hydrofluoric acid etching followed by silane) with respect to initial pull-out retention strength, retention after thermocycling, failure mode assessed by SEM, and surface wettability.

Based on these objectives, the following null hypotheses were tested:

(I) The different surface-conditioning protocols would not influence the pull-out retention strength of zirconia copings cemented to Ti-base abutments;
(II) Thermocycling would not affect the retention strength of the tested protocols;
(III) The evaluated surface-conditioning protocols would not influence the failure pattern observed after debonding; and
(IV) The tested protocols would not affect the wettability of the zirconia surface.

## 2. Materials and Methods

To fulfill the defined objectives, an in vitro experimental study was conducted in which several determinations were performed

### 2.1. Initial Pull-Out Retention Test

**Figure 1:**
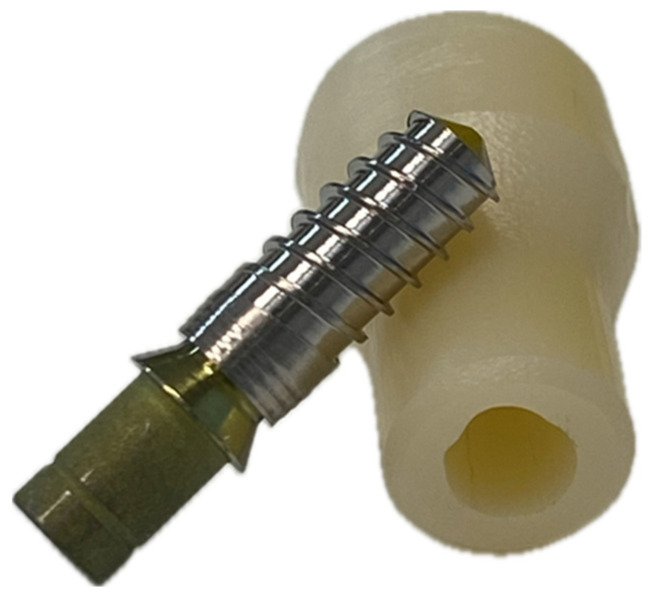
Detail of the milled and sintered monolithic zirconia copings and the Ti-base abutment screwed into VEGA + Klockner implants.

The experimental model comprised monolithic zirconia copings, milled and sintered, designed for cementation onto Ti-base abutments screwed with a 30 Ncm preload into internal-connection dental implants (VEGA+ Klockner; Soadco, Escaldes-Engordany, Andorra). The internal retentive surfaces replicated the typical geometry and thickness of zirconia crowns, while the occlusal portion was shaped to allow stable engagement with the traction arm of the universal testing machine (Figure 1).

A preliminary sample of 12 specimens per surface-treatment group was first analysed to estimate the variance of pull-out measurements. Based on these pilot data, a sample size calculation was performed assuming a significance level of α = 0.05, a statistical power of 80%, and the detection of a large effect size for a one-way ANOVA design (f = 0.40). Considering three independent experimental groups, the analysis indicated that a minimum of 21 specimens per group would be required to achieve adequate statistical power. Consequently, the main experiment included 21 zirconia copings per group. The calculation was implemented using R software version 4.5.3 (R Core Team, Vienna, Austria) with the pwr package for prospective power analysis.

The three treatment groups were:

- C group: untreated control receiving only silanization.
- AE group: hydrofluoric acid etching at 100 °C followed by silanization,
- APA group: airborne-particle abrasion with alumina followed by silanization,

In the AE group, the 21 copings were conditioned by internal etching with 9% hydrofluoric acid at 100 °C for 10 min using the Zir Etchant Cloud system (Medi 5, Incheon, Korea), following the manufacturer’s specifications. Silane was applied afterwards.

In the APA group, airborne-particle abrasion was performed using 110 µm alumina particles at 6 bar pressure and a 5 mm distance for 15 s with a microblaster (Airsonic Alu-Oxyd; Hager & Werken GmbH & Co. KG, Duisburg, Germany). Silanization was then applied as the final surface-conditioning step.

In the C (control) group, silanization was the only conditioning procedure performed on the zirconia surface. In contrast, in the AE and APA groups, silane was applied after their respective preliminary conditioning steps (acid etching or airborne-particle abrasion).

The silane agent (Anaxblend Zircon Bonder; Anaxdent, Stuttgart, Germany) was applied following a commonly used protocol for zirconia primers. A uniform layer was brushed onto the internal surface for 30 s, allowed to evaporate for 1 min, and dried with warm air to ensure solvent removal and monomer activation.

All copings were cemented by the same operator. The cementation procedure followed the manufacturer’s instructions using a self-adhesive resin cement (RelyX Unicem 2 Automix; 3M ESPE, Seefeld, Germany). Automix syringes were used to minimise mixing variability. Each coping was fully filled with cement and seated onto the Ti-base with firm manual pressure for 20 s, followed by axial loading of 5 kg for 10 min. Excess cement was removed after 30 min, and the marginal fit was visually verified to ensure complete seating without discrepancies. This cementation protocol aligns with methodologies reported in in vitro studies of similar design [5,18].

**Figure 2:**
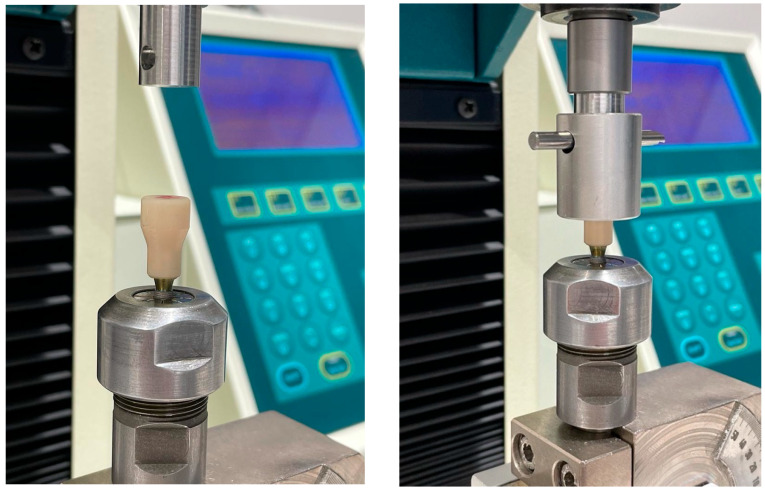
Image of a coping cemented onto the Ti-base abutment screwed into the implant and connected to the traction arm of the universal testing machine for the pull-out test.

After an additional 30 min, the initial pull-out test was performed. Each implant–Ti-base–coping assembly was mounted in a universal testing machine, and tensile load was applied at 1 mm/min until coping displacement occurred (Figure 2). The maximum force (N) required for debonding was recorded.

### 2.2. Pull-Out Retention Test After Thermocycling

A total of 30 new zirconia copings (n = 10 per surface-treatment group: AE, APA, C) were used. Surface treatments and cementation procedures were identical to those described above. The 30 specimens underwent 5000 thermocycles between water baths at 5 °C and 55 °C using a thermocycling machine (CM4; SODEMA S.L., Barcelona, Spain). Each cycle lasted 60 s, consisting of 20 s in each bath and 10 s transfer time. After thermocycling, pull-out tests were repeated under the same loading conditions as in the initial evaluation.

### 2.3. Scanning Electron Microscopy (SEM)

For each surface-treatment group, five copings retrieved after the initial pull-out test and five additional copings retrieved after thermocycling followed by pull-out testing were examined using scanning electron microscopy (SEM). The specimens selected for SEM observation were randomly chosen from the available samples within each group using a simple random selection procedure.

The objective was to determine the proportion of resin cement remaining on the titanium abutment versus the internal zirconia surface after debonding. Imaging was performed with a JEOL JSM-6400 microscope (JEOL Ltd., Tokyo, Japan). Cement distribution on both surfaces was quantified using ImageJ software version 4.0 (National Institutes of Health, Bethesda, MD, USA). The entire surface that was in contact with the cement was analyzed. The sample was rotated inside the sample holder of the scanning electron microscope, analyzing the quantity of each of the phases using backscattered electrons. The use of this technique allows gray tones to be distinguished by atomic weight and facilitates the identification of the different phases. The software automatically controlled the different gray tones to detect the surface covered by cement and the surface that was not covered.

This analysis aimed to identify the weakest component of the zirconia–cement–titanium assembly. Three failure patterns were considered:

- Predominant cement remaining on zirconia, indicating that the weakest interface was between the resin cement and the titanium abutment.
- Predominant cement remaining on titanium, indicating that the weakest interface was between the resin cement and the zirconia coping.
- Similar amounts of cement on both surfaces, suggesting primarily cohesive failure within the cement layer.

### 2.4. Wettability Assessment (Sessile Drop Method)

Nine zirconia discs (three per surface treatment group) were prepared for wettability analysis. The discs were milled and sintered under the same conditions as the copings, with a diameter of 5 mm and a thickness of 2 mm. Surfaces were polished with a fine-grain 1200-grit abrasive disc, washed with distilled water, methyl alcohol, and acetone, and dried with warm air to ensure complete dehydration. The contact angle was measured only on the treated surface.

Wettability was evaluated using the sessile drop method. A 1 µL droplet of deionized water was placed on each specimen and allowed to stabilize for 1 min before measurement. Contact angles were recorded using an OCA 11 goniometer (Dataphysics Instruments, Filderstadt, Germany), and the software automatically calculated the droplet contact angles. For each disc, nine consecutive measurements were obtained and averaged. Surface roughness effects were corrected using the Wenzel equation.

### 2.5. XRD

The monoclinic phase present in the zircona was studied and the possible degradation was analysed by X-ray diffraction using a Bruker diffractometer (Bruker AXS GmbH, Karlsruhe, Germany) with Cu K_a_ of 1.54 Å. The voltage, intensity and step-size applied were 40 kV, 20 mA and 0.02°.

### 2.6. Surface Roughness Analysis

Surface topography was evaluated using white-light interferometry microscopy (Wyko NT1100, Veeco Instruments Inc., Plainview, TX, USA). Five specimens from each experimental group (C, AE, and APA) were selected for analysis through a simple randomization procedure based on a random-number generator. For each specimen, seven measurements were taken at different locations on the surface, and the mean of these measurements was calculated to represent the roughness of that specimen. The analyzed surface area for all measurements was 459.9 × 604.4 μm^2^. Data processing was performed with Wyko Vision 232^TM^ software version 2.1 (Veeco Instruments Inc., Plainview, TX, USA). A Gaussian filter was applied to separate waviness and form from the surface texture, using a cut-off value of λc = 0.25 mm. The areal roughness parameters Sa (arithmetical mean height of the surface) and Sz (maximum height of the surface, defined as the sum of the largest peak height and the largest pit depth within the measured area). were calculated. The mean values obtained for each specimen were then used for statistical comparison among the experimental groups.

### 2.7. Statistical Analysis

Data normality was assessed using the Anderson–Darling test. Once normality was confirmed, a one-way analysis of variance (ANOVA) was performed to determine whether significant differences existed among the three surface-treatment groups. When the ANOVA indicated significant effects, pairwise comparisons were carried out using Tukey’s post hoc test. The significance level was set at α = 0.05. Statistical analyses were conducted using Minitab 20 (Minitab LLC, State College, PA, USA).

A sample size calculation was performed for the primary mechanical outcome of the study (initial pull-out retention test). The additional analyses included in the experimental design (SEM failure-pattern evaluation, wettability assessment, and surface roughness characterization) were conducted as complementary surface and mechanistic analyses intended to support the interpretation of the mechanical results and therefore followed sample sizes commonly reported in experimental biomaterials studies.

## 3. Results

Table 1 summarises the descriptive and inferential statistics for the initial and post-thermocycling pull-out tests, expressed in newtons (N), for the C, AE, and APA groups.

The results showed that the AE group (acid etching) exhibited the highest initial pull-out forces (N) required to debond the zirconia copings, with mean values 125% greater than those of the control group and 42.6% greater than those of the airborne-particle abrasion group, with statistically significant differences (F = 37.07; *p* < 0.001).

Similarly, after thermocycling and repetition of the mechanical test, the AE group again demonstrated the highest debonding forces, even exceeding its own initial values. In contrast, the control group displayed values nearly identical to its initial performance. Most notably, thermocycling was associated with a critical loss of debonding resistance in the APA group, which retained less than 50% of its initial pull-out strength.

**Figure 3:**
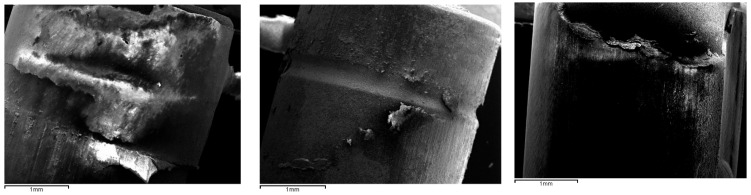
SEM analysis of the percentage of retained resin cement on the cemented surfaces after the pull-out tests. (**Left**): C group; (**Center**): AE group; (**Right**): APA group. It is worth noting the minimal amount of cement observed on the titanium surface of the Ti-base in the acid-etched specimen.

For the SEM analysis, ImageJ quantification allowed determination of the percentage of cement remaining on the zirconia or on the titanium wall of the Ti-base after both the initial and post-thermocycling pull-out tests (Figure 3).

The ImageJ findings were consistent with the mechanical test results. In the AE group (acid etching), the percentage of cement remaining adhered to the titanium surface was minimal and significantly lower than in the other two treatments, with statistically significant differences (*p* < 0.05). This suggests that, in this group, the weakest point of the bonded interface was the titanium surface. For the other two treatments, the percentages did not show statistically significant differences. Table 2 presents the percentage values obtained.

Table 3 shows the roughness values. It can be seen that there are statistically significant differences between the three groups with a *p* < 0.05. It can be seen that the particle projection treatments have greater roughness than the acid attacks and the control. In principle, greater roughness will have a larger specific surface area that will come into contact with the cement and provide greater fixation.

**Figure 4:**
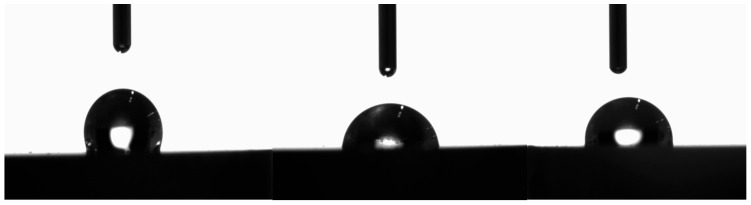
Image of the wettability test using a goniometer. (**Left**): C group; (**Center**): AE group; (**Right**): APA group.

Finally, regarding wettability, the C group showed a contact angle of 99.7° (SD 4.3) and the APA group 98.2° (SD 3.1), while the acid-etched AE group exhibited a lower value of 87.6° (SD 4.2). No statistically significant differences were found between the C and APA groups, but both differed significantly from the AE group (*p* < 0.05). These results indicate that high-temperature acid etching produces superior wettability of the zirconia surface (Figure 4), increasing the likelihood of effective contact between the resin cement and the Zr substrate.

**Figure 5:**
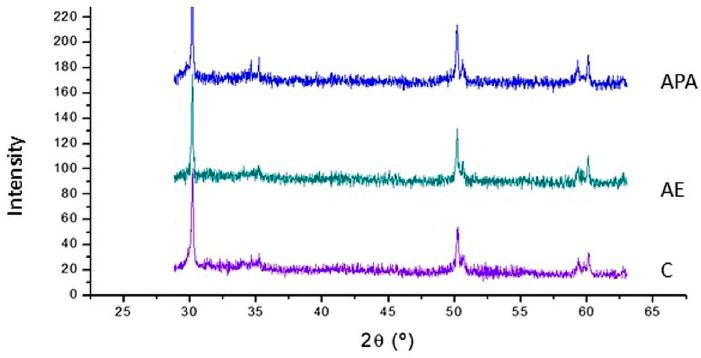
X-ray diffraction (XRD) patterns of zirconia surfaces after the different surface-conditioning treatments: control (C), acid etching (AE), and airborne-particle abrasion (APA). All groups show diffraction peaks corresponding to the tetragonal phase of zirconia. No additional peaks associated with monoclinic zirconia were detected after any of the treatments. For clarity, the diffractograms are vertically offset, and therefore the absolute intensity values should not be directly compared among groups.

Figure 5 shows the X-ray diffractometers with the different treatments to which the zirconia was subjected, showing the peaks of the tetragonal phase and no signs of the monoclinic phase in any case. Therefore, it can be concluded that the treatments do not affect the phases present in the zirconia.

## 4. Discussion

This in vitro experimental study aimed to evaluate the influence of three zirconia surface-conditioning protocols on the retention of zirconia copings cemented to titanium bases under mechanical tensile testing. The study also assessed the effect of thermocycling, which simulates the temperature and humidity fluctuations of the oral cavity, on the same dependent and independent variables, and provided a mechanistic interpretation of these outcomes through complementary SEM and wettability analyses. Finally, surface characterization of the treated specimens was completed by surface roughness analysis using white-light interferometry microscopy and X-ray diffraction (XRD) to assess the crystalline phase of zirconia after the different surface-conditioning treatments.

The findings showed that the high-temperature acid-etching protocol (AE group) produced significantly greater initial pull-out forces compared with both the control (C) and airborne-particle abrasion (APA) groups. The AE group exhibited mean retention values 125% higher than the control group and 42.6% higher than the APA group, confirming the strong effect of chemical conditioning on the zirconia–cement interface. After thermocycling, the superiority of the AE group remained evident, with retention forces exceeding their own initial measurements, while the APA group experienced a substantial reduction in retention, maintaining less than 50% of its initial strength. These results demonstrate that the mechanisms and durability of bonding differ markedly among the surface treatments evaluated.

Previous studies have indicated that mechanical or chemical surface treatments can enhance the retention of zirconia restorations on titanium abutments. These results are consistent with those of other in vitro studies [19], which also reported higher retentive strength in zirconia subjected to acid etching compared with alumina airborne-particle abrasion (n = 10), as well as increased micromechanical retention with higher concentrations or longer etching times.

A distinguishing feature of this study is the use of a high-temperature hydrofluoric acid etching protocol (9% HF at 100 °C for 10 min) within a controlled, closed etching system. While other investigations have explored the effects of hydrofluoric acid on zirconia, most were conducted at room temperature [17,19]. Under conventional conditions, zirconia is considered highly resistant to hydrofluoric acid; however, increasing the temperature of the etching solution may enhance its interaction with the zirconia surface and promote measurable surface modifications. In the present study, additional surface characterization was performed to better understand these effects. Surface roughness was evaluated by optical interferometry, showing that airborne-particle abrasion produced the highest roughness values (Sa, Sz), followed by the acid-etched group, whereas the untreated control group exhibited the lowest values. X-ray diffraction analysis did not reveal detectable phase transformation after any of the surface-conditioning protocols, indicating that none of the evaluated treatments induced tetragonal-to-monoclinic conversion of zirconia under the tested conditions. Interestingly, although airborne-particle abrasion produced the highest roughness values, the acid-etched group showed the highest retention values. This finding suggests that surface roughness alone may not fully explain the bonding behavior, and that additional factors such as surface energy and wettability may also play a relevant role. However, a limitation of the present study is that the analyses performed were mainly structural, and therefore the experimental design does not allow a detailed description of the adhesive mechanisms that may underlie the observed retention behavior. All samples were cemented using a self-adhesive resin cement, whose bonding mechanism relies on the interaction between phosphate monomers and zirconium oxide. The choice of cement was guided by previous in vitro studies demonstrating that self-adhesive resin cements maximise the retention of zirconia restorations [8]. Other variables that influence retention, such as abutment height [20], were not included in the present design; however, since this factor remained constant, the differences observed can be attributed primarily to the surface-conditioning method and are unlikely to represent confounding effects.

One of the most notable findings of this study is the significant influence of thermocycling on the reduction in retention in the APA group. These results are consistent with those reported in studies with similar experimental designs and objectives [21,22,23], which have shown that retention tends to decrease after aging when adhesion relies mainly on micromechanical surface irregularities, while chemically conditioned surfaces may exhibit more stable bonding behavior. One possible explanation is that airborne-particle abrasion can introduce surface defects in zirconia, such as microcracks or grain-boundary damage, which may compromise the stability of the cement interface under hydrothermal stress. In this context, a previous in vitro study reported that alumina abrasion applied after sintering may generate surface flaws—including lateral cracks, grooves, and plastic deformation—potentially facilitating water penetration and weakening the cement–zirconia interface during aging [24]. Qualitative observation of the treated surfaces also suggested differences in the morphology of the resulting topographies. The untreated control specimens exhibited surface marks consistent with the machining process, while the acid-etched surfaces appeared to present a more homogeneous micro-roughness pattern, and airborne-particle abrasion produced a more irregular and heterogeneous surface morphology. Although these observations were not quantitatively analyzed, they may help explain the greater reduction in retention observed in the APA group after thermocycling.

Furthermore, the relationship between crack propagation and grain size in abraded zirconia suggests that larger grains may allow more extensive crack development, thereby increasing fluid infiltration and accelerating aging. Although grain size was not directly evaluated in this study, this mechanism should be considered in future investigations. Additional studies comparing different particle sizes or pressures during abrasion, followed by thermocycling, may clarify how mechanical surface treatments influence long-term degradation of zirconia–cement interfaces. This relates to the in vitro study by Hee-Kyung Kim et al. [25], which suggests that larger grains may permit more extensive crack development, and their results indicate that the larger the grain size, the larger the crack.

A distinguishing feature of this work, compared with studies of similar design, is the inclusion of wettability and SEM analyses. These assessments were incorporated to better characterise the likelihood of enhanced cement-to-zirconia contact and to describe the associated failure patterns.

SEM analysis provided valuable complementary insight. Three failure modes were considered: cement predominantly on zirconia (indicating a weaker cement–titanium interface), cement predominantly on titanium (indicating a weaker cement–zirconia interface), or cohesive failure within the cement layer. In the AE group, nearly all cement remained on zirconia, indicating that the weakest interface was the cement–titanium junction and confirming the superior adhesion achieved by acid-etched zirconia. This pattern was entirely consistent with the mechanical results.

Although the SEM sample size was smaller than in other in vitro studies [26,27], the consistency of failure patterns strengthens the validity of these observations. These results reaffirm that chemical conditioning of zirconia combined with resin cement is the most reliable approach for preventing debonding.

Surface wettability results further support these findings. One in vitro study [28] demonstrated that airborne-particle abrasion increases micromechanical retention and surface wettability. In this study, the APA group showed slightly lower contact angles than the control, indicating a modest increase in surface energy. However, the most substantial improvement was observed in the AE group, which exhibited the lowest contact angle and thus the highest wettability. This elevated surface energy likely facilitates enhanced spreading and penetration of the resin cement, contributing to improved retention. Additionally, the more homogeneous surface topography produced by the acid-etching protocol may favor more uniform liquid spreading, which could further contribute to the improved wettability observed in this group. These results are consistent with another in vitro study [29], which reported that adhesion depends strongly on surface energy and that treatments increasing wettability significantly enhance bonding strength.

In addition to micromechanical retention associated with surface roughness, chemical interactions between zirconia surfaces and functional phosphate monomers in some resin cements have been described in the literature. Monomers such as MDP are known to interact with metal oxides, including zirconium oxide, potentially contributing to chemical adhesion. However, this study was not designed to investigate the specific contribution of these chemical mechanisms, as the cementation protocol was kept constant across all groups to isolate the effect of the different surface-conditioning treatments evaluated.

Although in vitro studies allow strict control of variables, pull-out tests cannot fully replicate intraoral conditions, where forces are multidirectional and cyclic. Artificial aging via thermocycling also cannot simulate mechanical fatigue, which may influence long-term adhesion. While these aspects represent limitations of this study, they are inherent to published studies of similar design and objectives.

Based on the results obtained, the null hypotheses regarding the influence of surface-conditioning protocols on pull-out retention strength, failure pattern, and surface wettability were rejected. In addition, the hypothesis that thermocycling would not affect the retention behavior of the tested protocols was also rejected, as significant differences were observed after artificial aging.

## 5. Conclusions

Within the limitations of this in vitro study, the following conclusions can be drawn:

High-temperature hydrofluoric acid etching followed by silanization significantly increased the pull-out retention strength of zirconia copings cemented to Ti-base abutments compared with airborne-particle abrasion plus silane and silane application alone.

After thermocycling, the acid-etched specimens maintained the highest retention values, whereas airborne-particle abrasion was associated with a marked reduction in debonding resistance.

SEM analysis showed that acid-etched zirconia surfaces predominantly retained cement on the zirconia side, indicating a stronger zirconia–cement interface.

Overall, high-temperature hydrofluoric acid etching followed by silanization proved to be the most effective surface-conditioning protocol among those tested, providing higher and more stable retention, more favourable failure modes, and improved wettability of zirconia copings cemented to Ti-base abutments.

## Figures and Tables

**Table 1:** Statistical analysis of the initial and thermocycled pull-out test results by group. Values are expressed as mean (SD). Different superscript letters indicate statistically significant differences among groups within each testing condition (*p* < 0.05, Tukey post hoc test).

Group	Initial Pull-Out Mean (sd)	Thermocycled Pull-Out Mean (sd)
C	205.3 (74.4) ^a^	207.99 (37.61) ^a^
AE	462.0 (112.8) ^c^	577.67 (50.89) ^b^
APA	324.0 (99.0) ^b^	155.81 (32.20) ^c^

**Table 2:** Percentage of residual cement adhered to the Ti-base walls after pull-out testing, before and after thermocycling, for the three experimental groups. Values are expressed as mean (SD). Different superscript letters indicate statistically significant differences among groups (Tukey post hoc test, *p* < 0.05).

Sample	Mean (SD) % of Cement on Ti-Base
Group C initial pull-out test	57.8% (12.3)% ^a^
Group C pull-out test after thermocycling	50.2% (12.3)% ^a^
Group AE initial pull-out test	8.1% (3.5)% ^b^
Group AE pull-out test after thermocycling	9.3% (4.1)% ^b^
Group APA initial pull-out test	50.5% (15.2)% ^a^
Group APA pull-out test after thermocycling	45.3% (19.8)% ^a^

**Table 3:** Surface roughness parameters obtained for the different experimental groups. Values are expressed as mean (SD). Different superscript letters indicate statistically significant differences among groups (Tukey post hoc test, *p* < 0.05).

Sample	Sa (µm) Mean (sd)	Sz (µm) Mean (sd)
Group C	0.12 (0.07) ^a^	0.56 (0.26) ^a^
Group AE	0.26 (0.09) ^b^	0.88 (0.22) ^b^
Group APA	0.91 (0.08) ^c^	1.11 (0.32) ^c^

## Data Availability

The original contributions presented in this study are included in the article. Further inquiries can be directed to the corresponding author.
